# Clinical Characteristics and Treatment Outcomes for Patients Infected with *Mycobacterium haemophilum*

**DOI:** 10.3201/eid2509.190430

**Published:** 2019-09

**Authors:** Pornboonya Nookeu, Nasikarn Angkasekwinai, Suporn Foongladda, Pakpoom Phoompoung

**Affiliations:** Faculty of Medicine, Siriraj Hospital, Bangkok, Thailand

**Keywords:** Mycobacterium haemophilum, bacteria, tuberculosis and other mycobacteria, nontuberculous mycobacteria, clinical characteristics, treatment outcomes, infection, Bangkok, Thailand

## Abstract

*Mycobacterium haemophilum* is a nontuberculous mycobacterium that can infect immunocompromised patients. Because of special conditions required for its culture, this bacterium is rarely reported and there are scarce data for long-term outcomes. We conducted a retrospective study at Siriraj Hospital, Bangkok, Thailand, during January 2012–September 2017. We studied 21 patients for which HIV infection was the most common concurrent condition. The most common organ involvement was skin and soft tissue (60%). Combination therapy with macrolides and fluoroquinolones resulted in a 60% cure rate for cutaneous infection; adding rifampin as a third drug for more severe cases resulted in modest (66%) cure rate. Efficacy of medical therapy in cutaneous, musculoskeletal, and ocular diseases was 80%, 50%, and 50%, respectively. All patients with central nervous system involvement showed treatment failures. Infections with *M. haemophilum* in HIV-infected patients were more likely to have central nervous system involvement and tended to have disseminated infections and less favorable outcomes.

*Mycobacterium haemophilum* is a nontuberculous mycobacterium that causes localized and disseminated infections in immunocompromised patients and rarely in immunocompetent patients (*1*). It is a slow-growing, aerobic, fastidious mycobacterium that requires heme-supplemented culture medium and low temperatures of 30°C–32°C for optimal growth (*2*). Because of the special conditions required for culture, it is frequently not isolated because of use of inappropriate techniques, and thus is rarely reported in the medical literature.

The most common clinical manifestation of infection in adult patients is cutaneous disease (*3*,*4*), either localized or as part of disseminated disease that occurs mainly in severely immunocompromised patients, such as those infected with HIV, those with autoimmune disease, or those who have undergone solid organ or stem cell transplantation (*5*–*10*). Thus, infection with *M. haemophilum* should be suspected in immunocompromised patients who have unexplained skin lesions and are smear positive for acid-fast bacilli, but show negative results for routine mycobacterial culture.

There is no current standardized guideline for optimal management of patients infected with *M. haemophilum*. Furthermore, the long-term outcome of this infection has not been well documented. The purpose of this study was to determine clinical characteristics, treatment, and long-term outcomes for infections with *M. haemophilum*.

## Methods

 We conducted a retrospective cohort study at Siriraj Hospital, the largest academic hospital in Bangkok, Thailand, during January 2012–September 2017. We identified all patients who were given a diagnosis of *M. haemophilum* infection by culture or molecular methods. Specimens of all types underwent smear microscopic analysis by using auramine–rhodamine staining and mycobacterial culture by using Lowenstein-Jensen solid medium and liquid medium containing mycobacteria growth indicator. All specimens were incubated at 35°C, and those from skin, bone, and joint were also incubated at 30°C. We performed species identification by using the INNO-LiPA Mycobacteria Version 2 Assay (Innogenetics [now Fujiregio], https://www.fujirebio-europe.com). We reviewed baseline demographics, clinical characteristics, microbiological data, antimicrobial and surgical treatment, and clinical outcome. All patients were followed up for >1 year after diagnosis. This study was approved by the institutional review board committee at Siriraj Hospital (Chart of Accounts no. Si 630/2017).

## Results

A total of 21 patients were included in this study; 67% were women (median age 53 years, range 25–73 years). All 21 patients were immunocompromised. The most common concurrent condition was HIV infection (8 patients, 38%), followed by systemic lupus erythematosus (5 patients, 23.8%), γ-interferon autoantibody (2 patients, 9.5%), kidney transplantation (2 patients, 9.5%), diabetes mellitus (2 patients, 9.5%), ankylosing spondylitis (1 patient), and nephrotic syndrome (1 patient).

All HIV-infected patients except 1 had CD4 cell counts <200 cells/mm^3^. Among non–HIV-infected patients, those with systemic lupus erythematosus, kidney transplantation, and nephrotic syndrome received corticosteroids or other immunosuppressive agents.

**Figure 1:**
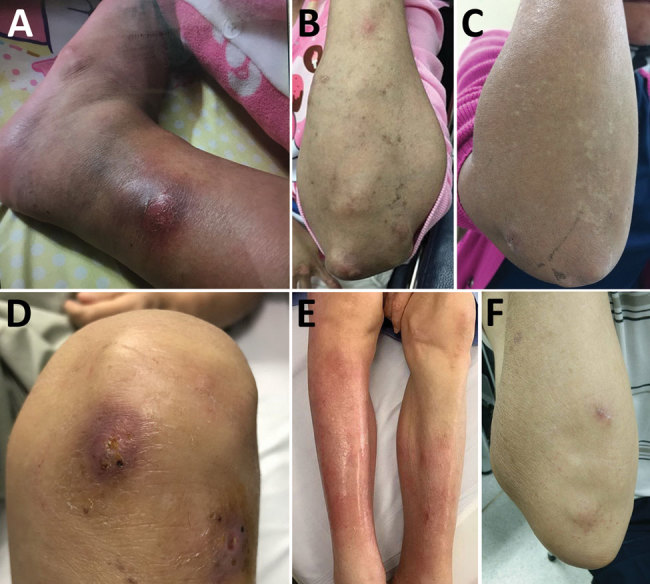
Cutaneous manifestation in non–HIV-infected patients infected with *Mycobacterium haemophilum*, Bangkok, Thailand. A) Patient 9, B) patient 11, C) patient 12, D) patient 16, E) patient 17, F) patient 21.

**Figure 2:**
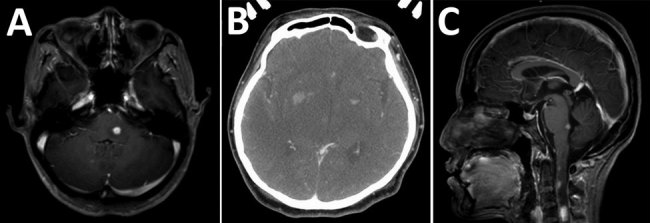
Imaging of brain and spine of 3 patients infected with *Mycobacterium haemophilum* who had involvement of the central nervous system, Bangkok, Thailand. A) Patient 1, axial T1-weighted magnetic resonance imaging scan with gadolinium showing enhanced nodule at left dorsal pons. B) Patient 2, axial contrast-enhanced computed tomography scan showing hypodensity lesions in both thalami and nodular enhancement at the bilateral basal ganglia. C) Patient 3, sagittal T1-weighted magnetic resonance imaging scan with gadolinium showing multiple enhancing nodules at dorsal pons and upper cervical cord.

The most common organ involved was skin and soft tissue (13 patients), followed by bone and joint (3 patients), central nervous system (CNS) (3 patients), eye (2 patients), and lymph nodes (1 patient). Two patients (1 with CNS involvement [no. 1] and 1 with bone and joint infection [no. 14]) had concomitant mycobacteremia. The most common cutaneous manifestation was an erythematous nodule that commonly occurred on the extensor surface of elbows, legs, and the auricular area (Figure 1). Of 7 skin biopsy specimens, granulomatous inflammation was the most common pathologic finding. Three patients who had CNS involvement had advanced HIV disease and CD4 cell counts <50 cells/mm^3^. We obtained computed tomography (CT) and magnetic resonance imaging findings for 3 patients with CNS involvement (Figure 2).

We did not perform antimicrobial drug susceptibility testing on any bacterial isolate because of a failure of growth in solid medium. A total of 20/21 patients were treated with a combination of antimycobacterial agents. For 19 patients whose outcomes were available, 11 patients were cured, 1 patient improved with ongoing antimicrobial drug treatment, 3 patients required surgical excision after failure of medical therapy, 3 patients had a relapse of their infection after treatment discontinuation, and 1 patient died from disseminated disease after 1 month of therapy (Table).

**Table:** Clinical characteristics and treatment for 21 patients infected with *Mycobacterium haemophilum* Bangkok, Thailand*

Patient no.	Age, y/sex	Disease or condition, CD4 cell count/mm^3^	Clinical manifestation	Site of positive culture	Surgical treatment	Drug treatment	Treatment duration, mo†	Outcome
1	25/F	AIDS, 17	Brain abscesses, septicemia	Blood	None	AZM, LVX, EMB	1	Died
2	35/F	AIDS, 12	Brain abscesses	Brain tissue	None	NA	NA	Lost to follow-up
3	35F	AIDS, 40	Myelitis	Spinal cord tissue	None	INH, RIF, PZA, EMB, CLR, AMK	2	Treatment failure
4	29/M	AIDS, 14	Skin nodule (left popliteal fossa)	Skin	None	AZM, LVX, RIF	NA	Lost to follow-up
5	52/M	AIDS, 6	Plague (right hand)	Skin	None	CLR, CIP, RIF	6	Cured
6	46/F	AIDS, 190	Chronic ulcer (left foot)	Pus	None	MFX	3	Cured
7	36/F	AIDS, 12	Auricular abscess	Pus	None	AZM, LVX, RIF	12	Cured
8	53/F	HIV+, 657	Preauricular abscess	Pus	I and D	CLR, CIP	12	Cured
9	25/F	SLE	Chronic wound and osteomyelitis (right ankle), olecranon bursitis	Bone	Debridement	IMI, AMK; then AZM, CIP, RIF	6 (1.5/4.5)	Relapsed
10	39/F	SLE	Tenosynovitis (right index finger)	Pus	Debridement	AMK; then CLR, LVX	6 (2/4)	Cured
11	57/F	SLE	Skin nodules (both elbows)	Skin	None	CLR, CIP, RIF	6	Cured
12	47/F	SLE, dermatomyositis	Skin nodules (both elbows)	Skin	None	IMI, AMK; then AZM, CIP, RIF	6 (0.5/5.5)	Cured
13	39/F	SLE, DM	Skin abscess (right ankle)	Pus	I and D	AMK; then CLR, LVX, DCS	12 (2/10)	Cured
14	69/M	IFN-γ autoantibody	Septicemia, spondylodiscitis	Blood	None	CLR, LVX, RIF	24	Relapsed
15	73/F	IFN-γ autoantibody	Lymphadenitis (right cervical node)	Lymph node	None	IMI, CLR; then CLR, CIP, SXT	12	Cured
16	60/F	Kidney transplant	Skin nodules (both arms/legs), septic arthritis (right ankle)	Pus	Debridement	AMK, CLR, LVX, LZD; then MFX, AZM, RIF, LZD	11 (1/10)	Improved
17	58/F	Kidney transplant	Plague (both legs)	Skin	None	IMI, AMK, CLR; then AZM, LVX, RIF	12 (1/11)	Relapsed
18	65/F	DM, A1C 6.7%	Scleritis and keratitis	Sclera	Enucleation	IMI, CLR, LVX, RIF, LZD	4	Treatment failure
19	65/M	DM, A1C 13.3%	Endophthalmitis	Vitreous fluid	None	IMI, AMK, LVX; then AZM, RIF, DOX	12 (0.5/11.5)	Cured
20	53/M	Ankylosing spondylitis	Skin nodules (right elbow)	Skin	Surgical excision	CLR, LVX	6	Treatment failure
21	48/M	Nephrotic syndrome	Skin nodules (right elbow)	Skin	None	CLR, CIP, RIF	12	Cured

*AMK, amikacin; AZM, azithromycin; A1C, hemoglobin A1C; CIP, ciprofloxacin; CLR, clarithromycin; DCS, d-cycloserine, DM, diabetes mellitus; DOX, doxycycline; EMB, ethambutol; I and D, incision and drainage; IFN-γ, interferon-γ; IMI, imipenem; INH, isoniazid; LVX, levofloxacin; LZD, linezolid; MFX, moxifloxacin; NA, not available; PZA, pyrazinamide; RIF, rifampin; SLE, systemic lupus erythematosus; SXT, sulfamethoxazole/trimethoprim; +, positive. †Values in parentheses are durations for each drug group.

The success rate of medical therapy for cutaneous infection was 80%. However, this rate was lower (50%) for bone, joint, and ocular infections. All patients with CNS diseases and involvement showed treatment failures.

The most commonly used regimen included a combination of macrolides and fluoroquinolones (3 patients, 14.3%) or these combined regimens with rifampin (9 patients, 42.9%). Combination therapy with macrolides and fluoroquinolones resulted in a success rate of 60% for treatment of cutaneous infection. Use of rifampin as the third drug for more severe cases also resulted in a modest (66%) success rate. Sulfamethoxazole/trimethoprim, doxycycline, and cycloserine were also replaced with rifampin, which showed clinically successful results. For 11 patients in whom antimicrobial drugs could be discontinued, the median duration of treatment was 12 months (range 3–12 months for skin and soft tissue infections, 6 months for bone and joint infections, and 12 months for lymphadenitis and eye infections).

The patient who died of disseminated *M. haemophilum* infection was a 25-year-old man who was given a new diagnosis of infection with HIV and had a CD4 cell count of 17 cells/mm^3^. He had diplopia for 1 month and a low-grade fever. Physical examination showed multiple left-sided cranial nerve palsies (V [trigeminal], VI [abducens], and VII [facial]) and lower motor neuron lesions. Magnetic resonance imaging of the brain showed multiple, abnormal, high-signal-intensity lesions on T2-weighted imaging with gadolinium, as well as nodular enhancement of the left dorsal pons, right ventral pons, mid pons, left cerebellar peduncle, and medulla (Figure 2, panel A). Examination of cerebrospinal fluid showed standard results; hemoculture grew *M. haemophilum*. He was given levofloxacin, azithromycin, and ethambutol. However, his clinical condition deteriorated rapidly. Right hemiparesis then developed and he became stuporous. He died from acute respiratory failure secondary to aspiration pneumonia.

When we compared HIV-infected and non–HIV-infected patients, HIV-infected patients were younger (median age 36 years vs. 57 years; p = 0.017), more likely to have disseminated infection (37.5% vs. 15.4%; p = 0.325), more likely to have CNS involvement (37.5% vs. 0%; p = 0.042), and more likely to have a less favorable prognosis (50% vs. 77%; p = 0.38).

## Discussion

We report 21 cases of *M. haemophilum* infection over a 6-year period at the largest academic hospital in Thailand. *M. haemophilum* commonly causes infection in immunocompromised persons. Advanced HIV infection remains the most common immunocompromised condition associated with this infection, as reported (*1*,*3*). Approximately 60% of patients have skin and soft tissue involvement, and the most commonly involved areas are the extensor surfaces of the extremities and auricular regions, which could be explained by the predilection of the organism for body areas with lower temperatures.

Although CNS infection with *M. haemophilum* is extremely rare and only a few case-patients have been reported (*11*–*13*), we identified CNS involvement in 3 of 21 case-patients in our study. All had advanced HIV disease: 2 patients had multiple brain abscesses, which was similar to those previously described, and 1 patient had myelitis. A total of 2 of 3 previous case reports of CNS involvement in patients with *M. haemophilum* infection were from Thailand and in HIV-infected patients (*11*,*12*), whereas the 2 largest case series (23 and 15 cases) reported from the United States found no cases of CNS involvement (*3*,*4*). Further study is needed to determine whether genetic or environmental factors will influence clinical manifestations of *M. haemophilum* infection.

Treatment with a combination of fluoroquinolones, rifampin, and macrolides is suggested for treating *M. haemophilum* infection (*14*). However, our study demonstrated that 2 antimycobacterial agents (macrolides and fluoroquinolones) were successfully used for patients with isolated cutaneous diseases. Conversely, surgical resection might be needed for some case-patients, such as those who showed treatment failure or those in which there was CNS involvement. Because of poor penetration of the CNS by these antimicrobial agents, patients who had mycobacterial infections with CNS involvement are often associated with poorer outcomes (*15*,*16*). Two previous case reports of persons with CNS disease were successfully treated with surgical excision in combination with antimicrobial drugs, although there were residual neurologic deficits (*11*,*13*). Another study reported a case-patient who did not respond to medical therapy alone and subsequently died (*12*).

Treatment for *M. haemophilum* infection should last ≈3–12 months and should be tailored on the basis of severity of disease and immunocompromised conditions. Isolated cutaneous disease usually responds well to shorter duration of therapy (3–6 months), and CNS infections, bone and joint infections, and disseminated disease usually require longer therapy (12 months) (*1*). Relapse cases have been rarely reported (*17*), and accounted for just 4% in the largest case series (*1*). However, our study showed a higher relapse rate (14%); therefore, we suggest that careful monitoring after discontinuation of treatment is warranted.

One limitation of our study was that no antimicrobial susceptibility testing was available because all culture-positive cases were detected in liquid medium. However, no data support the correlation of in vitro susceptibility testing and treatment response for this type of infection.

*M. haemophilum* infections should be suspected in immunocompromised patients who have unexplained cutaneous lesions, especially at auricular or extensor surfaces of extremities, who are smear positive for acid-fast bacilli, but show negative results for routine mycobacterial culture. Combination antimycobacterial therapy should be given for >3 months and extended to 12 months depending on the site of isolation. CNS involvement might occur more commonly than previously believed and is associated with worse outcome. Relapses are not uncommon; therefore, clinical monitoring after discontinuation of treatment is warranted.
